# Deletion of Tgfβ signal in activated microglia prolongs hypoxia‐induced retinal neovascularization enhancing Igf1 expression and retinal leukostasis

**DOI:** 10.1002/glia.24218

**Published:** 2022-05-25

**Authors:** Ayumi Usui‐Ouchi, Kevin Eade, Sarah Giles, Yoichiro Ideguchi, Yasuo Ouchi, Edith Aguilar, Guoqin Wei, Kyle V. Marra, Rebecca B. Berlow, Martin Friedlander

**Affiliations:** ^1^ Department of Molecular Medicine The Scripps Research Institute La Jolla California USA; ^2^ Department of Ophthalmology Juntendo University Urayasu Hospital Chiba Japan; ^3^ The Lowy Medical Research Institute La Jolla California USA; ^4^ Gene Expression Laboratory Salk Institute for Biological Studies La Jolla California USA; ^5^ Department of Regenerative Medicine Chiba University Graduate School of Medicine Chiba Japan; ^6^ Department of Bioengineering University of California San Diego La Jolla California USA; ^7^ Department of Integrative Structural and Computational Biology The Scripps Research Institute La Jolla California USA

**Keywords:** a mouse model of oxygen‐induced retinopathy, angiogenesis, diabetic retinopathy, hypoxia, insulin like growth factor 1, ischemic retinopathy, microglia, neovascularization, retinopathy of prematurity, transforming growth factor

## Abstract

Retinal neovascularization (NV) is the major cause of severe visual impairment in patients with ischemic eye diseases. While it is known that retinal microglia contribute to both physiological and pathological angiogenesis, the molecular mechanisms by which these glia regulate pathological NV have not been fully elucidated. In this study, we utilized a retinal microglia‐specific Transforming Growth Factor‐β (Tgfβ) receptor knock out mouse model and human iPSC‐derived microglia to examine the role of Tgfβ signaling in activated microglia during retinal NV. Using a tamoxifen‐inducible, microglia‐specific Tgfβ receptor type 2 (Tgfβr2) knockout mouse [Tgfβr2 KO (ΔMG)] we show that Tgfβ signaling in microglia actively represses leukostasis in retinal vessels. Furthermore, we show that Tgfβ signaling represses expression of the pro‐angiogenic factor, Insulin‐like growth factor 1 (Igf1), independent of Vegf regulation. Using the mouse model of oxygen‐induced retinopathy (OIR) we show that Tgfβ signaling in activated microglia plays a role in hypoxia‐induced NV where a loss in Tgfβ signaling microglia exacerbates and prolongs retinal NV in OIR. Using human iPSC‐derived microglia cells in an in vitro assay, we validate the role of Transforming Growth Factor‐β1 (Tgfβ1) in regulating Igf1 expression in hypoxic conditions. Finally, we show that Tgfβ signaling in microglia is essential for microglial homeostasis and that the disruption of Tgfβ signaling in microglia exacerbates retinal NV in OIR by promoting leukostasis and Igf1 expression.

## Main Points

- Blocking Tgfβ signaling in retinal microglia induces activation and worsens OIR neovascularization by promoting leukostasis and Igf1 upregulation
- Tgfβ1 regulates Igf1 expression in microglia, rescuing hypoxia‐induced Igf1 upregulation in microglia

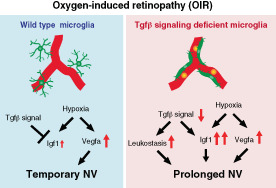

## 1 INTRODUCTION

Retinal neovascularization (NV) is the major cause of severe visual impairment in patients with ischemic or inflammatory ocular diseases such as diabetic retinopathy, retinal vein occlusion, uveitis and retinopathy of prematurity (Usui et al., 2015). Vascular Endothelial Growth Factor (VEGF) plays a pivotal role in the development of pathological NV; drugs that inhibit this pro‐angiogenic cytokine have been widely used to treat retinal neovascular diseases (Fogli et al., 2018; Witmer et al., 2003). While intravitreal anti‐VEGF injection represents a major breakthrough for the treatment of retinal neovascular diseases, not all patients respond to anti‐VEGF agents (Ashraf et al., 2016; Ip et al., 2015; Writing Committee for the Diabetic Retinopathy Clinical Research et al., 2015). Moreover, there are safety issues associated with repeated intravitreal administration of anti‐VEGF agents, especially for at‐risk patients with diabetes, cardio‐ and cerebrovascular diseases, or premature babies who are vulnerable to modulation of crucial trophic factors (Falavarjani & Nguyen, 2013; Usui‐Ouchi & Friedlander, 2019). Understanding VEGF‐independent mechanisms of retinal NV and their role in retinal neovascular disease is critical for developing additional complimentary or alternative therapeutic strategies.

Numerous studies have shown that dysregulation of the VEGF‐independent pro‐angiogenic factor Insulin Growth Factor 1 (Igf1) is associated with pathological NV in proliferative diabetic retinopathy or retinopathy of prematurity (Boulton et al., 1997; Haurigot et al., 2009; Hellstrom et al., 2001; Kondo et al., 2003; Meyer‐Schwickerath et al., 1993; Ruberte et al., 2004; Smith et al., 1999; Wilkinson‐Berka et al., 2006). In patients with proliferative diabetic retinopathy, vitreous levels of IGF1 are increased (Boulton et al., 1997; Grant et al., 1986; Meyer‐Schwickerath et al., 1993). In mouse OIR models, endothelial cell specific IGF1 receptor KO mice show reduced retinal NV (Kondo et al., 2003), and overexpression of IGF1 in the retina results in phenotypic changes similar to those of diabetic retinopathy (pericyte loss, capillary basement membrane thickness, inner retinal microaneurysms, and neovessels) (Ruberte et al., 2004). IGF1 plays a key role in pathological NV in retina, however the origin and regulation of IGF1 has not been fully characterized.

Microglia are the resident immune cells in the retina localized to the outer and inner plexiform layers and superficial plexus, engaging in surveillance and maintenance of retinal synapses (Lee et al., 2008; Wang et al., 2016). Under pathological conditions such as retinal degeneration, neovascularization and aging, microglia are activated and migrate into the affected sites where they respond to inflammation by up‐regulationg phagocytic activity and expression of inflammatory cytokines (Ma et al., 2009; Usui‐Ouchi et al., 2020; Zhao et al., 2015). Activated and mis‐localized retinal microglia are a common hallmark of various retinal degenerative, inflammatory and angiogenic diseases (Silverman & Wong, 2018). In ischemic retinopathy, activated microglia are found in the central avascular zone prior to neovascularization (Fischer et al., 2011; Vessey et al., 2011). In the OIR mouse model of ischemic retinopathy, ablation of microglia can rescue NV (Kubota et al., 2009) suggesting a key role in pathological NV. However, their role appears to be independent of VEGF activation (Boeck et al., 2020). The mechanism by which microglia are activated and promote NV is not understood.

Tgfβ1 has previously been shown to be a potent immunoregulatory factor for microglia in vivo and in vitro where the loss of Tgfβ signaling results in the increase of microglia activation (Brionne et al., 2003; Butovsky et al., 2014; Ma et al., 2019; Spittau et al., 2013; Zoller et al., 2018). Tgfβ‐signaling is propagated by binding of Tgfβ to Tgfβ receptor type 2 (Trfbr2) that phosphorylates the Tgfβ receptor type 1 (Tgfbr1)(Wrana et al., 1994; Yamashita et al., 1994). Pan‐ocular deletion of Tgfβ signaling can also cause common changes observed in proliferative diabetic retinopathy including pericyte loss, microaneurysms, leaky capillaries, and retinal hemorrhages (Braunger et al., 2015). Targeted ablation of Tgfbr2 in retinal microglia promotes activation causing a neuroinflammatory response and choroidal NV (W. Ma et al., 2019). However, the role of Tgfβ signaling in microglia on retinal NV and the mechanisms that regulate microglia function during ischemia are not understood.

Here, we investigate the role of Tgfβ signaling in the activation of microglia and demonstrate that it can induce VEGF‐independent NV pathways through regulation of Igf1. Using the OIR mouse model of ischemic retinopathy, targeted ablation of Tgfbr2 in microglia, and human iPSC derived microglia we demonstrate that hypoxia in the retina regulates microglial activation, the expression of chemoattractant chemokines, leukostasis, and Igf1 dependent NV through the repression of Tgfβ signaling in microglia. These results provide insight into the mechanism of microglial activation under ischemic retina and the role they play in the formation of pathological NV.

## 2 MATERIALS AND METHODS

### 2.1 Mice and animal experimental procedures

All animal protocols were approved by the IACUC committee at The Scripps Research Institute, La Jolla, California. All animals received food and water ad libitum. C57BL6 mice and Balb/c mice were obtained from The Scripps Research Institute animal facility. Chemokine (C‐X3‐C motif) receptor 1 (Cx3cr1)^Cre−ERT^ mice expressing tamoxifen‐inducible Cre recombinase (The Jackson Laboratory, #021160) (Parkhurst et al., 2013) were crossed with mice possessing loxP sites that flank exon 4 of the Tgfbr2 (Tgfbr2^flox/flox^, The Jackson Laboratory, #012603) (Leveen et al., 2002) to generate Cx3cr1^Cre−ERT^; Tgfbr2^flox/flox^. To induce Cx3cr1‐Cre recombination, 100 μg of tamoxifen (Sigma‐Aldrich, T5648)/cone oil solution was administered to Cx3cr1^Cre−ERT^; Tgfbr2^flox/flox^ and control littermates, Tgfbr2^flox/flox^ (Control) subcutaneously once a day from P9 to P14 and for OIR P4 to P6 and P12 to P14 to avoid the oxygen level fluctuation in chamber from P7 to P12. Oxygen‐induced retinopathy (OIR) was induced as previously described (Murinello et al., 2019; Smith et al., 1994). Postnatal day 7 (P7) pups and their mothers were exposed to 75% oxygen in a hyperoxia chamber (BioSpherix ProOx P110) for 5 days and returned to room air at P12. Mice were euthanized by cervical dislocation at varying time points, as indicated in the results and figure legends.

### 2.2 Cell and cell culture

The human induced pluripotent stem cell (hiPSC) line used was derived from peripheral blood mononuclear cells from a female. Reprogramming was performed by the Harvard iPS core facility using sendai virus for reprogramming factor delivery. All cell lines were obtained with verified normal karyotype and contamination‐free. hiPSC were maintained on Matrigel (BD Biosciences) coated plates with mTeSR1 medium (STEMCELL Technologies). Cells were passaged every 3–4 days at approximately 80% confluence. Colonies containing clearly visible differentiated cells were marked and mechanically removed before passaging. Microglia precursors were generated as previously described (Haenseler et al., 2017; van Wilgenburg et al., 2013). The embryoid bodies (EBs) are formed using Aggrewells (STEMCELL Technologies), cultured with bone morphogenetic protein 4 (BMP4), vascular endothelial growth factor (VEGF), and stem cell factor (SCF), then plated into T175 flasks with Interleukin‐3 and macrophage colony‐stimulating factor (M‐CSF). After 4 weeks, microglia precursors emerged into the supernatant. It was previously revealed that their ontogeny is MYB‐independent primitive myeloid cells, which is same ontogeny as microglia (Buchrieser et al., 2017). hiPS derived Microglia precursors (pMG) were plated into 12 well plates containing X‐VIVO15 with 100 ng/ml M‐CSF, 2 mM Glutamax, 100 U/ml penicillin, and 100 μg/ml streptomycin for further in vitro assays. The cells were stimulated with human recombinant Tgfβ1 (Peprotech, 100‐21), 10 μM of SB525334 (Selleckchem, S1476), 200 μM of DMOG (Millipore sigma, D3695), then cell culture supernatant and cells were stored at −80°C for following qPCR and ELISA assays.

### 2.3 Immunohistochemistry of whole‐mount retinas

Enucleated eyes were placed in 4% paraformaldehyde (PFA) for 1 h. After fixation, the cornea, the lenses, the sclera, choroid, and the vitreous were removed and the retinas were laid flat with four radial relaxing incisions. Retinas were incubated in blocking buffer (PBS with 10% fetal bovine serum, 10% normal goat serum, and 0.2% Triton X‐100) for 2 h at 4°C, following by an overnight incubation with primary antibodies in blocking buffer at 4°C. Tissue specimens were then washed and incubated with the corresponding Alexa fluorescent‐conjugated secondary antibodies (Thermo Fisher) for 3 h. Retinas were washed in PBS and mounted with ProLong Diamond Antifade mounting medium (Thermo Fisher Scientific, P36965). Primary antibodies targeting IBA1 (1:500; FUJIFILM, 019‐19741), and NG2 (1:200; Millipore, AB5320) were used. Fluorescent‐conjugated isolectin Griffonia Simplicifolia IB‐4 (GS‐lectin) (1:200; Thermo Fisher Scientific, I21413, I32450) was also used for labeling endothelial cells. For detecting hypoxic cells in vivo, 60 mg/kg bodyweight pimonidazole hydrochloride (Hypoxyprobe‐1™ kit, hpi), diluted in PBS was administered by intraperitoneal injection 1 h prior to euthanasia. Enucleated eyes were processed as above and stained with FITC anti‐pimonidazole antibody (1:100, Hypoxyprobe‐1™ kit, hpi). All images were acquired with a confocal laser scanning microscope (LSM 710, Zeiss) and processed with the ZEN 2010 software (Zeiss).

### 2.4 Retinal microglia isolation by flow cytometry

A postnatal neural dissociation kit (Miltenyi, 130‐092‐628) was used to prepare a single cell suspension from mouse retinas. Cells were centrifuged at 150*g* for 5 min at 4°C. The digested tissue was resuspended in 100 μl of 4% FBS in PBS containing an FITC antibody to CD11b (1:100; BioLegend, 101206) and PE antibody to Gr‐1(1:100; BD Biosciences, 553128) and incubated for 20 min on ice. The cells were washed and suspended with 1 ml of 4% FBS/PBS containing DAPI (1:2000; Thermo Fisher Scientific, 62248) and DRAQ5 (1:5000; Cell signaling, 40845) for exclusion of dead cells and debris. We used clone RB6‐8C5 for Gr‐1 antibody because it reacts with a common epitope on Ly6‐G and Ly6‐C to eliminate blood born monocytes and granulocytes. We did not use CD45 antibody to detect CD45^low^ fraction as microglia population because CD45 expression in Tgfbr2‐ablated microglia is upregulated transforming to activated form as previously shown (Ma et al., 2019). Labeled retinal microglia (CD11b positive and Gr‐1 negative in DAPI negative and DRAQ5 positive cells) were isolated by fluorescence‐activated cell sorting (FACS) (MoFlo Astrios EQ; Beckman Coulter) at the Scripps Flow Cytometry Core Facility. Sorted cells were resuspend in 350ul of RLT buffer from RNeasy Micro Kit (QIAGEN) and stored at −80°C.

### 2.5 RNA isolation and real‐time PCR

For whole retina and culture cells, single retinas were collected in 500 μl of Trizol and total RNA was isolated using a PureLink RNA Mini Kit (Thermo Fisher Scientific) according to manufacturer's instructions. Seven hundred and fifty nanograms of RNA was used for RT‐qPCR using a high‐capacity cDNA reverse transcription kit (Thermo Fisher Scientific). For flow‐sorted cells, total RNA was isolated from sorted cells using the RNeasy Micro Kit (QIAGEN) and reverse transcribed using Maxima First Strand cDNA Synthesis Kit for RT‐qPCR (Thermo Scientific). qPCR was performed using Power‐up SYBR™ Green PCR Master Mix (Thermo Fisher Scientific) and primers on a Quantstudio 5 Real‐Time PCR System (Thermo Fisher Scientific). β‐actin (*Actb*) was used as the reference gene for all experiments. Levels of mRNA expression were normalized to those in controls as determined using the comparative CT (ΔΔ*CT*) method. Primer sequences are listed in Table S1.

### 2.6 Enzyme‐linked immunosorbent assay (ELISA)

Forty‐eight hours after Tgfβ1 supplementation to hiPS derived pMG, cell culture supernatants were assayed for ELISA assay to detect the protein level of IGF1 using the Human IGF‐1 Quantikine ELISA kit (R&D systems) according to the manufacturer's protocol.

### 2.7 Lectin labelling of adherent retinal leukocytes

The retinal vasculature and adherent leukocytes were imaged by perfusion labeling with TRITC‐conjugated Concanavalin A (Con A) lectin (Vector Laboratories), as described previously (Joussen et al., 2001; Okunuki et al., 2019). Briefly, after deep anesthesia, the chest cavity was opened and a 27‐ gauge cannula was inserted into the left ventricle. Mice were then perfused through the left ventricle first using 5 ml of PBS, followed by fixation with 1% PFA (5 ml), 5 ml of TRITC‐conjugated Con A (20 μg/ml in PBS), and 5 ml of PBS. The eyes were then fixed in 4% PFA for an hour, and the retinas were flat‐mounted. The total number of TRITC positive adherent leukocytes in the retinal vessels was counted.

### 2.8 Quantification and statistical analysis

For OIR, the percentage of the area of NV and vaso‐obliteration (VO) in OIR retinas was automatically quantified using deep learning segmentation software available at http://oirseg.org (Xiao et al., 2017). All statistical tests were performed in GraphPad Prism v8 (GraphPad Software, Inc). Data comparisons between two groups were performed using unpaired two‐tailed Student *t*‐tests. Data comparisons between multiple groups were performed with one‐way ANOVA with Tukey's correction. Statistical tests used for each experiment are specified in the figure legends. Data are represented as mean ± SEM. A p value of *p* < .05 was considered significant.

### 2.9 Study approval

All animal protocols were approved by the IACUC committee at The Scripps Research Institute, La Jolla, California, and all federal animal experimentation guidelines were adhered to.

## 3 RESULTS

### 3.1 Tgfβbr2 deficient microglia transform to activated status

**FIGURE 1:**
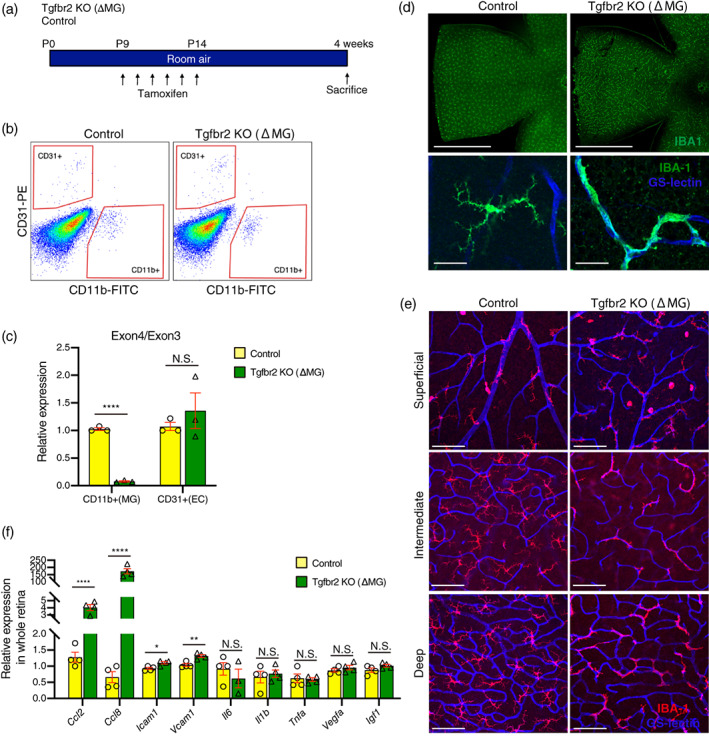
Tgfβbr2 deficient microglia transform to an activated state. (a) Schematic showing the protocol was used to generate data for Figure 1. (b) the representative flow sorting chart for isolating retinal Cd31 positive endothelial cells and Cd11b positive microglia from control (left) and Tgfβbr2 KO (ΔMG) (right) mice 4 weeks after tamoxifen administration. (c) the ratio of exon3 to exon4 expression in Tgfbr2 was determined by qPCR in Cd11b + microglia (MG) or Cd31+ endothelial cells (EC). The expression of exon4 was specifically inhibited in microglia (n = 3 each). Data are mean ± SEM. *p* Values were calculated using a two‐tailed Student's *t*‐test, ****p < .0001. N.S., not significant. (d) Retinal flatmount stained with IBA1 to visualize microglia 4 weeks after tamoxifen administration to control and Tgfβbr2 KO (ΔMG) mice. Microglia throughout the retina were observed to be transformed and activated in Tgfβbr2 KO (ΔMG) mice. Upper panel: Low magnification (scale bars = 1 mm). Lower panel: High magnification (scale bars = 50 μm). (e) the relation between retinal vessels and microglia at each vessel layer 4 weeks after tamoxifen administration to control and Tgfβbr2 KO (ΔMG) mice (scale bars = 100 μm). (f) mRNA expression in retinas from control or Tgfβbr2 KO (ΔMG) mice (n = 4 each). Data are mean ± SEM. *p* Values were calculated using a multiple *t*‐test, *p < .05, ** *p* < .01, **** *p* < .0001, N.S. = not significant.

Tgfβ1 has previously been shown to be a potent immunoregulatory factor for microglia in vivo and in vitro where the loss of Tgfβ signaling results in the increase of microglia activation (Brionne et al., 2003; Butovsky et al., 2014; Ma et al., 2019; Spittau et al., 2013; Zoller et al., 2018). To investigate the role of Tgfβ signaling in microglia activation and NV we used microglia specific Tgfbr2 knockout mice (Tgfbr2 KO (ΔMG)) with Cx3cr1^Cre−ERT^; Tgfbr2^flox/flox^. As Tgfbr2 is highly expressed in both endothelial cells and microglia in mouse retina (Braunger et al., 2015; Ma et al., 2019), we first confirmed whether exon 4 of Tgfbr2 is excised specifically in microglia compared to endothelial cells in Tgfbr2 KO (ΔMG) retinas 4 weeks after tamoxifen injection (Figure 1a). We performed a qPCR analysis of mRNA isolated from flow‐sorted CD11b + retinal microglia and showed a marked reduction in exon 4 containing transcripts in Tgfbr2 KO (ΔMG) relative to exon 3, but not in tamoxifen‐administered control mice, while the exon 4/3 ratio in CD31 positive endothelial cells was not changed in Tgfbr2 KO (ΔMG) compared to control mice (Figure 1b,c). We examined microglial morphology and distribution 4 weeks after tamoxifen injection in Tgfbr2 KO (ΔMG) and we found that Tgfbr2 deficient microglia tightly adhered to retinal vessels in all three layers: superficial plexus, intermediate plexus in the inner plexiform layer, and deep plexus in the outer plexiform layer. Tgfbr2 deficient microglia had shorter processes with less branching and a larger soma, while microglia from control mice did not adhere to vessels and had fine, long processes (Figure 1d,e). To examine if the activation of microglia in Tgfbr2 KO (ΔMG) promotes retinal inflammation, we measured the expression of inflammatory cytokines, chemokines, and adhesion molecules in whole retina samples of Tgfbr2 KO (ΔMG) and control mice by qPCR. The expression of monocyte chemoattractants Ccl2 and Ccl8 were highly upregulated, and adhesion molecules Icam1 and Vcam1 were slightly but significantly upregulated in Tgfbr2 KO (ΔMG) retina compared to control retina. The expression of other proinflammatory cytokines Il6, Il1b, and Tnf and proangiogenic factors Vegfa and Igf1 were not changed in Tgfbr2 KO (ΔMG) retinas (Figure 1f).

### 3.2 Tgfβr2 deficient microglia promoted leukostasis via enhancing chemoattractant

**FIGURE 2:**
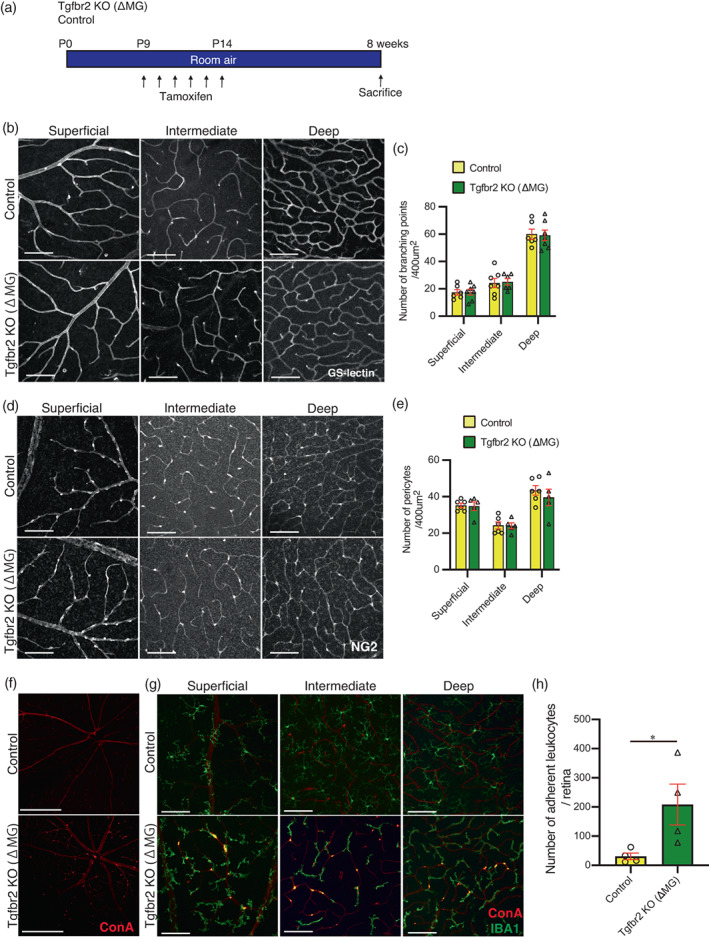
Tgfβ signal deletion in microglia did not affect structure of vessels and vascular development but, promoted the adhesion of leukocytes via enhancing chemoattractant. (a) The schematic shows the protocol that was used to generate data for this figure. (b) GS‐lectin labeled retinal vessels at superficial, intermediate, and deep plexus 8 weeks after tamoxifen injection from P9 to P14. Scale bars = 100 μm. (f) TRITC‐conjugated Concanavalin a (con a) lectin labeled leukocytes adhering to retinal capillary endothelium. Scale bars = 500 μm. (b) the number of branching points per 400 μm^2^ was evaluated (control *n* = 6, Tgfβbr2 KO (ΔMG) *n* = 7). (c) NG2 positive pericytes at superficial, intermediate, and deep plexus 8 weeks after daily tamoxifen injections on P9 to P14 (scale bars = 100 μm). (e) the number of NG2 positive pericytes per 400 μm^2^ was evaluated (control *n* = 6, Tgfβbr2 KO (ΔMG) *n* = 5). (f) TRITC‐conjugated Concanavalin a (con a) lectin labeled leukocytes adhering to retinal capillary endothelium (scale bars = 500 μm). (g) Adherent leukocytes to retinal capillaries are visualized by TRITC‐conjugated con a lectin at each layer. There are more leukocytes in all layers in Tgfβbr2 KO (ΔMG) mice compared to control (scale bars = 100 μm). (h) the number of adherent leukocytes labeled with con a lectin in Tgfβbr2 KO (ΔMG) mice was higher than those observed in control mice (*n* = 4 each). Data are mean ± SEM. *p* Values were calculated using a two‐tailed Student's *t*‐test, *p < .05.

To determine the consequence of the tight adherence of retinal microglia to retinal vessels in Tgfβbr2 KO (ΔMG), we assessed vascular development and morphology in Tgfβr2 KO (ΔMG) retina 8 weeks after tamoxifen injection (Figure 2a). We first evaluated the gross morphology of retinal vessels by measuring the number of branching points and found no difference in Tgfbr2 KO (ΔMG) compared to control (Figure 2b‐c). Next, we evaluated the number of pericytes along retinal vasculature because pericyte loss is early pathological sign of diabetic retinopathy (Engerman, 1989). However, we found no significant difference in Tgfβbr2 KO (ΔMG) compared to control (Figure 2d–e). Since we found that the expression of monocyte chemoattractants Ccl2 and Ccl8 were highly upregulated and adhesion molecules Icam1 and Vcam1 were slightly but significantly upregulated in Tgfβbr2 KO (ΔMG) retina (Figure 1f), we next examined leukocyte recruitment and adhesion to retinal vessels. To examine the leukocyte adhesion to retinal vessels in microglia specific Tgfβbr2 knockouts, we performed lectin labeling of adherent leukocytes. The retinal flatmount from Tgfbr2 KO (ΔMG) after lectin perfusion showed adherent leukocytes in the vessels while this was not observed in the majority of control retinal flatmounts (Figure 2f–g). The adhered leukocytes expressed IBA1 suggesting that those were circulating monocytes. The number of lectin labeled adherent leukocytes was significantly higher in the retina of Tgfbr2 KO (ΔMG) than in that of control (Figure 2h). Taken together, Tgfbr2 knock out microglia which adhered to vessels promote leukocyte adhesion via enhancing chemoattractant, without affecting retinal vascular development or morphology.

### 3.3 Igf1 expression was highly upregulated in Tgfβbr2 deficient microglia

**FIGURE 3:**
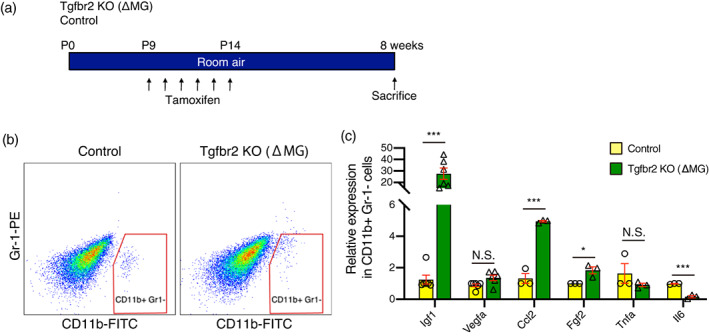
The proangiogenic factor Igf1 was highly upregulated in Tgfβbr2 deficient microglia. (a) Schematic showing the protocol used to generate data shown in this figure. (b) Representative flow charts for Cd11b+ Gr‐1‐ microglia sorting in control (left) and Tgfbr2 KO (ΔMG) (right) mice. (c) the expression of Igf1, Vegfa, Ccl2, Fgf2, Tnfa, and Il6 in retinal microglia of Tgfbr2 KO (ΔMG) or control mice was determined by qPCR (Igf1, and Vegfa: *n* = 6, Ccl2, Fgf2, Tnfa, and Il6: *n* = 3 each). Data are mean ± SEM. *p*‐Values were calculated using a two‐tailed Student's *t*‐test, *p < .05, ***p < .001. N.S., not significant.

Next, we examined if Tgfβbr2 deficient microglia themselves have pro‐angiogenic effects. We sorted microglia out from retinal cells by flow cytometry 8 weeks after tamoxifen injection into Tgfbr2 KO (ΔMG) and control (Figure 3a) mice. To eliminate circulating and adherent leukocytes, CD11b positive and Gr‐1 negative fractions were sorted out from the retina of Tgfbr2 KO (ΔMG) and control. The expression of pro‐angiogenic genes in the fraction were evaluated by qPCR (Figure 3b). We found that the expression of Igf1, Ccl2, and Fgf2 in microglia from Tgfbr2 KO (ΔMG) were significantly higher than microglia from controls (Figure 3c). Among those genes, the expression of Igf1 in microglia from Tgfβbr2 (ΔMG) was particularly impacted, increasing by 20–50‐fold. This demonstrates that knocking out Tgfβbr2 leads to significantly elevated levels of Igf1, suggesting that Tgfβ signaling is a powerful inhibitor of Igf1 expression in microglia.

### 3.4 The expression of Tgfβ receptors was downregulated and the expression of Igf1 was upregulated specifically in hypoxic microglia of wild type C57BL6 OIR

**FIGURE 4:**
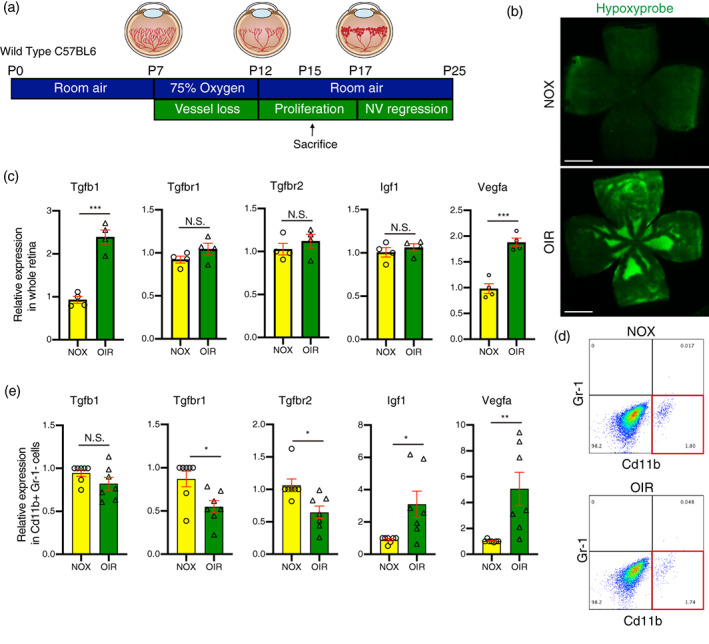
The expression of Tgfβ receptors was suppressed, and Igf1 upregulated, in hypoxic microglia of oxygen‐induced retinopathy (OIR) mice. (a) Schematic showing the protocol used to generate data for the OIR experiment in this figure. (b) The hypoxic retina in P15 OIR mice was detected by Hypoxyprobe‐1™ (green) (scale bars = 1 mm). (c) The representative flow‐sorting scatter stained by Gr‐1 and Cd11b. (d) The expression of Tgfβb1, Tgfβbr1, Tgfβbr2, Igf1, and Vegfa in Cd11b+ and Gr‐1‐ microglia sorted out from P15 OIR and normoxic mice was determined by qPCR (*n* = 7 each). Data are mean ± SEM. *p* Values were calculated using a two‐tailed Student's *t*‐test, *p < .05, **p < .01. N.S., not significant. (e) The expression of Tgfβb1, Tgfβbr1, Tgfβbr2, Igf1, and Vegfa in whole retina from P15 OIR and normoxic mice was determined by qPCR (*n* = 4 each). Data are mean ± SEM. *p* Values were calculated using a two‐tailed Student's *t*‐test, ***p < .001. N.S., not significant.

Numerous studies have shown that Igf1 is associated with pathological NV in proliferative diabetic retinopathy or retinopathy of prematurity (Boulton et al., 1997; Haurigot et al., 2009; Hellstrom et al., 2001; Kondo et al., 2003; Meyer‐Schwickerath et al., 1993; Ruberte et al., 2004; Smith et al., 1999; Wilkinson‐Berka et al., 2006). Since we have found that Tgfβ signaling regulates Igf1 expression in microglia, we next examined the potential regulation of Igf1 in microglia by Tgfβ signaling as a retinal angiogenic factor using OIR, the well‐established mouse model for studying hypoxia‐induced retinal NV (Smith et al., 1994) (Figure 4a). To investigate the interaction between Tgfβ signal and microglial activation under hypoxia, as well as the relevance of Igf1 in microglia to hypoxia‐induced NV, we examined the expression of Tgfβ receptors and Igf1 in the microglia of hypoxic retinas.

We examined the hypoxia response of the OIR retina at P15, using hypoxyprobe labelling, an indicator of hypoxic conditions in cells. We chose P15 because NV tufts emerge just after P15 preceded by hypoxic response in OIR retina. Elevated hypoxyprobe labeling shows hypoxia in avascular areas of the OIR retina (Figure 4b). We next determined if Tgfβb and Igf1 are altered in hypoxic retinas using qPCR. In whole retina, although the expression of Tgfβ1 and Vegfa was significantly upregulated in OIR retina compared to normoxia retina, the expression of Igf1, Tgfβbr1, and Tgfβbr2, were comparable between whole retina from OIR and normoxia (Figure 4c). However, the flow‐sorted Cd11b positive/Gr‐1 negative microglia from C57BL6 OIR retina showed significantly higher expression of Igf1 as well as Vegfa compared to normoxia control retinas. OIR microglia also showed lower expression of Tgfβbr1 and Tgfβbr2, suggesting that the expression of Igf1, and Tgfβb receptors, Tgfβbr1 and Tgfβbr2 were uniquely altered in hypoxic microglia in OIR (Figure 4d,e).

### 3.5 The blockage of Tgfβ signal in microglia exacerbated and prolonged activation of pathological NV in the OIR

**FIGURE 5:**
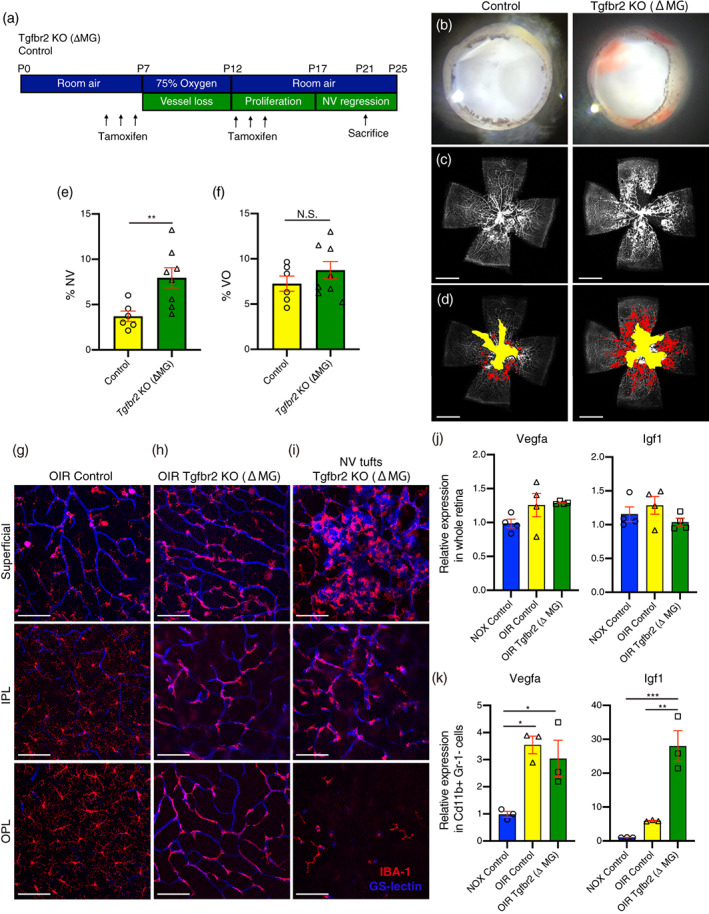
The blockage of Tgfβ signal in microglia exacerbate and prolong activation of pathological neovascularization (NV) in oxygen induced retinopathy (OIR). (a) Schematic showing the protocol used to generate data shown in the OIR experiment. The area of NV and vaso‐obliteration (VO) were analyzed at P21 when the OIR usually shows NV regression after its peak at P17. (b) Representative retinal cups from P21 OIR. Retinal hemorrhage was detected in retinas from Tgfβbr2 KO (ΔMG) mice. (c) Retinal flat mount at P21 OIR stained with fluorescein‐conjugated isolectin Griffonia Simplicifolia IB‐4 (GS‐lectin) for retinal vessels. (d) The area of NV pseudocolored in red and the area of VO pseudocolored in yellow (scale bars = 1 mm). (e, f) The quantification of NV (d) and VO (f) areas (control *n* = 6, Tgfbr2 KO (ΔMG) *n* = 8). Data are mean ± SEM. *p* Values were calculated using a two‐tailed Student's *t*‐test, **p < .01, N.S., not significant. (g–i) Microglia and retinal vessels were visualized by immunostaining of IBA1 (red) and GS‐lectin (blue), respectively, in control (g), vascularized parts of Tgfβbr2 KO (ΔMG) (h), and neovascular tuft of Tgfβbr2 KO (ΔMG) (i) at each retinal layer. Microglia from Tgfβbr2 KO (ΔMG) tightly adhered to superficial, intermediate, deep plexus as well as neovascular tufts. IPL, inner plexiform layer, OPL, outer plexiform layer, NV tufts, neovascular tufts. Scale bars = 100 μm. (j) The expression of Vegfa and Igf1 in whole retina from normoxic (NOX) control, OIR control, and Tgfbr2 KO (ΔMG) OIR (*n* = 4 each). Data are mean ± SEM. (k) The expression of Vegfa and Igf1 in Cd11b+ Gr‐1‐ microglial cells from NOX control, OIR control, and Tgfβbr2 KO (ΔMG) OIR. Mice (*n* = 3 each). Data are mean ± SEM. *p* Values were calculated using ordinary one‐way ANOVA and Tukey's multiple comparison test. *p < .05, **p < .01, ***p < .001.

Next, we examined if the deletion of Tgfβ signal in microglia exacerbated hypoxia induced retinal NVs in OIR. In the OIR model the retina usually shows NV regression and vessel regrowth in the area of vaso‐obliteration (VO) following P17, when VO is at its peak (Ma & Li, 2021) (Figure 5a). To investigate the role of microglial Tgfβ signaling in hypoxia‐induced retinal NV, we utilized the OIR model in Tgfβbr2 KO (ΔMG) mice. At P21, when the OIR usually shows NV regression (Figure 5a), we detected retinal hemorrhaging and a significant increase in NV in Tgfβbr2 (ΔMG) compared to wildtype OIR controls (Figure 5a–e), whereas the area of VO was unchanged between the two groups. As with Tgfβbr2 KO (ΔMG) in normoxia, the retinal microglia in Tgfβbr2 KO (ΔMG) in OIR at p21 were also altered and adhered to vessels in all three layers (Superficial, IPL, and OPL) (Figure 5g,h). At the region of NV tufts that develop in OIR, a large number of activated microglia adhered to NV (Figure 5i), suggesting that the Tgfβbr2 deficient microglia exacerbated and prolonged activation of pathological NV in OIR. We next examined the expression level of Vegfa and Igf1 in retina of Tgfβbr2 KO (ΔMG) OIR compared to control OIR, and control normoxic mice at p21. Unlike the OIR retina at p15, the expression of Vegfa is no longer upregulated in OIR at p21 in the whole retina. In normoxic controls, OIR, and Tgfβbr2 KO (ΔMG) OIR, Vegfa and Igf1 were comparable between each group (Figure 5j). Similar to OIR at p15 when we isolated Cd11b positive/Gr‐1 negative microglia using flow sorting we found that both Vegfa and Igf1 expression were still increased in OIR compared to control (Figure 5k). However only Igf1 expression is exacerbated in Tgfβbr2 KO (ΔMG) OIR compared to wildtype OIR. Vegfa expression was not changed between Tgfβbr2 KO (ΔMG) OIR and control OIR microglia (Figure 5k).

### 3.6 Tgfβ1 regulates Igf1 expression in human iPS cell‐derived microglia precursor cells

To validate the regulation of Igf1 expression by Tgfβ signal and hypoxia in microglia, we utilized human microglia precursor cells derived from human iPS cells (pMG) according to the well‐established protocol from Haenseler et al. (2017). The hiPSC derived microglia precursor cells from this protocol have previously been validated to develop in an MYB‐independent, RUNX1‐, and PU.1‐dependent fashion, consistent with in vivo microglia development (Buchrieser et al., 2017; Prinz & Priller, 2014). We validated pMG using qPCR, flow cytometry, and immunohistochemistry. These cells highly expressed microglia specific markers P2ry12 and Tmem119 as well as macrophage/microglia marker Cd11b by qPCR compared to HUVEC or PBMC (Figure S1a). Flow cytometry showed expression of CD45 (a pan‐leukocyte marker), CD14 (a component of the receptor for bacterial lipopolysaccharide), CX3CR1 (fractalkine receptor), and Cd11b on pMGs as previously reported (Figure S1b). Immunocytochemistry also showed that CD45, CD14, IBA1 (microglia/macrophage‐specific calcium binding protein), P2RY12, and PU.1 (myeloid transcription factor) were also expressed in pMG (Figure S1c).

**FIGURE 6:**
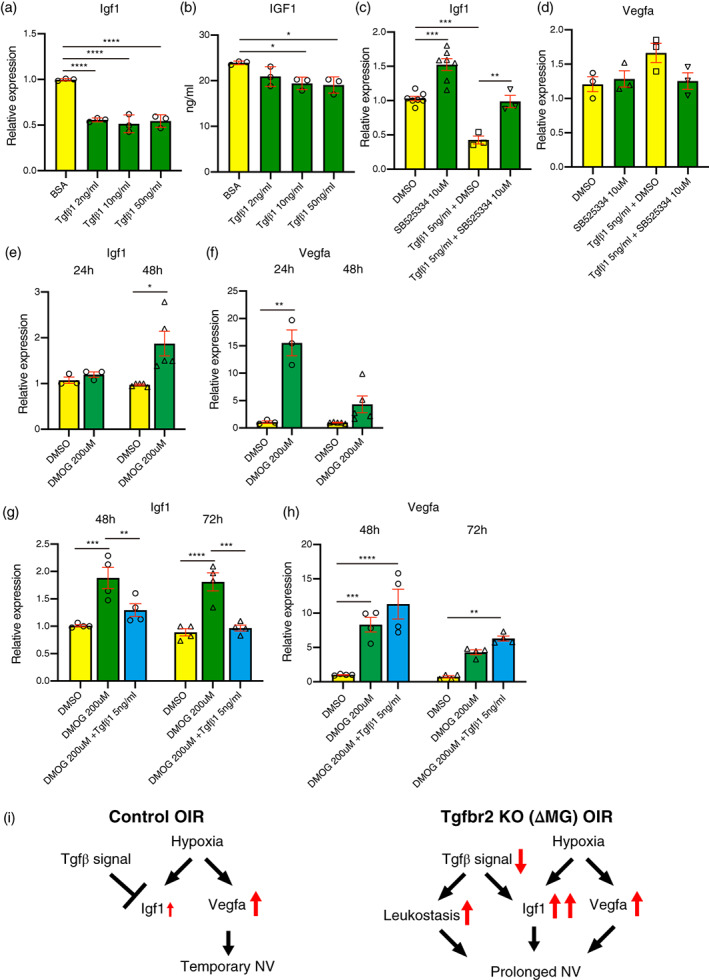
Tgfβ1 regulates Igf1 expression in human iPSCs derived microglia. (a) Igf1 expression was downregulated with 2, 10, 50 ng/ml of human recombinant Tgfβ1 24 h after supplementation (*n* = 3). Data are mean ± SEM. *p* Values were calculated using ordinary one‐way ANOVA and Tukey's multiple comparison test. ****p < .0001. (b) the protein concentration of IGF1 in the supernatant of human iPSCs derived microglia was determined by ELISA and downregulated after human recombinant Tgfβ1 supplementation (*n* = 3). Data are mean ± SEM. *p* Values were calculated using ordinary one‐way ANOVA and Tukey's multiple comparison test. *p < .05. (c, d) Relative Igf1 (c) and Vegfa (d) expression levels were determined by qPCR 48 h after supplementation. 10 μM of SB525334, Tgfbr2 inhibitor induced Igf1 expression and SB525334 inhibited Tgfβ1 induced Igf1 downregulation (DMSO and SB525334: *n* = 7, Tgfβ1 + DMSO and Tgfβ1+ SB525334: *n* = 3). Data are mean ± SEM. *p* Values were calculated using ordinary one‐way ANOVA and Tukey's multiple comparison test. **p < .01, *** *p* < .001. (e, f) 200 μM of dimethyloxallyl glycine (DMOG) was supplemented to induce chemical hypoxia in hiPSC derived microglia and the expression of Igf1 (e) and Vegfa (f) were evaluated at 24 and 48 h after the treatments (24 h: *n* = 3 each, 48 h: *n* = 5 each) data are mean ± SEM. *p* Values were calculated using a two‐tailed Student's *t*‐test, *p < .05, **p < .01. (g, h) 5 ng/ml Tgfβ1 rescued DMOG induced Igf1 (g) upregulation, but not Vegfa (h) upregulation at 48 and 72 h after the treatments (*n* = 4 each). Data are mean ± SEM. *p* Values were calculated using ordinary one‐way ANOVA and Tukey's multiple comparison test. **p < .01, ***p < .001, ****p < .0001. (i) The schema shows the relationship between Tgfβ signal and hypoxia‐induced retinal neovascularization in retinal microglia from control OIR or Tgfbr2 KO (ΔMG) OIR.

We next validated the suppression of Igf1 by Tgfβ signaling in human pMGs using Tgfβ ligand and Tgfβ signaling inhibitors. Twenty four hours following the addition of human recombinant Tgfβ1 to pMG culture media, the expression of Igf1 mRNA in pMG was significantly suppressed as expected (Figure 6a). We subsequently confirmed that IGF1 protein secreted into media was also suppressed by human recombinant Tgfβ1 (Figure 6b). Conversely, addition of the Tgfβbr2 inhibitor, SB525334, to pMGs increased Igf1 expression and was sufficient to rescue Igf1 suppression in the presence of Tgfβ1 (Figure 6c). Vegfa expression in pMG was not changed by Tgfβ1or SB525334 (Figure 6d). Next, to determine if hypoxic conditions could regulate the expression of Igf1 in pMG, we utilized the hypoxia mimetic Dimethyloxalylglycine (DMOG), which stabilizes hypoxia inducible factor‐1a (HIF‐1α). Vegfa, which is a direct target of HIF‐1α, showed a large and significant increase in expression in pMGs following 24 h of DMOG treatment which tapered off by 48 h (Figure 6f). However, Igf1 expression in pMG was not upregulated following 24 h of DMOG treatment and only showed a significant increase in expression at 48 h indicating that it is not directly induced by HIF‐1α (Figure 6e). Next, we looked at the interaction between hypoxia and Tgfβ signaling on Igf1 regulation and found that human recombinant Tgfβ1 rescued hypoxia induced Igf1 upregulation (Figure 6g), but had no effect on hypoxia induced Vegfa upregulation (Figure 6h).

## 4 DISCUSSION

We have demonstrated that Tgfβ signal in microglia played a role in regulating microglia homeostasis; inhibiting it resulted in exacerbating pathological NV through the upregulation of Igf1 in a mouse model of ischemic retinopathy.

Microglia are resident immune cells in the central nervous system. Under healthy conditions, retinal microglia are mainly localized in the outer and inner plexiform layer and superficial plexus where they engage in surveillance and maintenance of retinal synapses (Lee et al., 2008; Wang et al., 2016). However, under pathological conditions such as retinal degeneration, neovascularization and aging, microglia activate and migrate into the sites where those pathological changes occur. They also localize to damaged photoreceptors, RPE, and retinal or choroidal NV (Ma et al., 2009; Usui‐Ouchi et al., 2020; Zhao et al., 2015). Given this association between activated/primed microglia and neurovasculodegenerative diseases, it is necessary to better understand activation mechanisms in microglia.

Tgfβ signaling has pleiotropic effects in various tissues on cell survival and inflammation (Travis & Sheppard, 2014). In mammals, the Tgfβ family consists of three members, Tgfβ1, Tgfβ2, and Tgfβ3, and all isoforms can be detected in various types of ocular cells including retinal neurons, retinal pigment epithelium, blood vessels, and microglia (Tosi et al., 2018). Tgfβ receptors are also broadly expressed in different retinal cell types, specifically, in retinal microglia and endothelial cells (Ma et al., 2019; Obata et al., 1999). Deletion of pan‐ocular Tgfβ signaling leads to proliferative diabetic retinopathic changes such as pericyte loss, the formation of abundant microaneurysms, leaky capillaries, and retinal hemorrhages (Braunger et al., 2015). Tgfβ1 has previously been described as a potent immunoregulatory factor for cerebral microglia in vivo and in vitro (Brionne et al., 2003; Makwana et al., 2007; Spittau et al., 2013; Zoller et al., 2018). As for retinal microglia, Ma et al. demonstrated that ablation of Tgfbr2 in retinal microglia induced their activation as well as the promotion of pathological microglia gene expression profiles resulting in secondary Muller cell gliosis, neuronal apoptosis, and abnormal synaptic transmission (Ma et al., 2019). We have also detected activated pathological microglia in our microglia specific Tgfbr2 knockout mice although we used a different Cre induction protocol with Tamoxifen. Ma and colleagues also reported that Tgfbr2 deficient microglia demonstrated an exaggerated response to laser‐induced choroidal NV (Ma et al., 2019). However, the mechanism whereby Tgfβ signaling in microglia led to choroidal NV formation was not well defined.

Activated microglia cells are found in the central avascular zone of retinas with ischemic retinopathy (Fischer et al., 2011; Vessey et al., 2011). Pharmacological microglia depletion rescues pathological NV in OIR (Kubota et al., 2009), indicating that retinal microglia may be involved in retinal NV in hypoxic retinas. Moreover, Boeck et al. recently demonstrated, using cell‐specific reporter mice, that microglia are the predominant myeloid cell population in areas of retinal NV in OIR. Macrophages rarely appear in these areas. Furthermore, activated retinal microglia alter their transcriptional profile and exhibit considerable proliferative ability (Boeck et al., 2020). However, the mechanism by which microglia become activated and promote NV under hypoxia has not been defined. In this study, we report the influence of retinal microglial Tgfβ signaling on retinal NV and its mechanism during the hypoxic response.

We have shown microglia in the avascular area of OIR are hypoxic and those cells express lower Tgfbrs and higher Igf1 than those in normoxic healthy retina. This suggests that lacking Tgfβ signal and Igf1 upregulation in microglia could exaggerate Vegfa induced pro‐angiogenic response under hypoxia. Using the immortalized rodent microglia cell line BV‐2, Yin et al. showed hypoxic microglia produce Igf1, increasing their pro‐angiogenic capacity (Yin et al., 2017). We demonstrated that chemical‐induced hypoxia upregulated Igf1expression. Moreover, the upregulation of Igf1 was also rescued by Tgfβ1 supplementation, suggesting that Tgfβ1 regulates hypoxia‐induced Igf1 upregulation in microglia.

We also report that Tgfbr2 ablation in microglia exacerbated pathological NV in OIR. We found two remarkable changes that could exacerbate pathological NV: (1) an increase in retinal leukostasis in retinal capillaries through production of chemoattractant factors; and (2) an increase in Igf1 expression in microglia. Retinal leukostasis indicates an increase in leukocyte recruitment and adhesion to the retinal capillary endothelium. Retinal leukostasis can lead to blood‐retinal barrier breakdown, capillary occlusion, and amplification of the inflammatory response in various retinal diseases such as diabetic retinopathy, ischemic retinopathy, and uveitis (Eshaq et al., 2017; Tarr et al., 2013; Tsujikawa & Ogura, 2012). Dysregulated microglia caused by Tgfbr2 ablation were closely adherent to retinal vessels and produced chemoattractant factors such as Ccl2 and Ccl8, chemokines that can lead to the recruitment of circulating monocytes, promoting pathological NV.

Numerous studies have shown that Igf1 is associated with pathological NV in proliferative diabetic retinopathy or retinopathy of prematurity (Boulton et al., 1997; Haurigot et al., 2009; Hellstrom et al., 2001; Kondo et al., 2003; Meyer‐Schwickerath et al., 1993; Ruberte et al., 2004; Smith et al., 1999; Wilkinson‐Berka et al., 2006). For example, IGF1 is required for maximal VEGF‐dependent NV via the IGF1 receptor and MAPK activation in OIR, and Igf1 knockout mice had impaired retinal vascular growth despite normal VEGF level (Smith et al., 1999). Jacobo et al demonstrated that IGF1 stabilizes endothelial cell tubes and retinal neovessels that form in response to VEGF, mediating prolonged activation of Erk, which antagonizes lysophosphatidic acid (LPA)‐driven regression (Jacobo & Kazlauskas, 2015). Endothelial cell specific IGF1 receptor KO mice show reduced retinal NV in OIR (Kondo et al., 2003). On the other hand, overexpression of IGF1 in the retina results in changes similar to those of diabetic retinopathy (pericyte loss, capillary basement membrane thickness, inner retinal microaneurysms, and neovessels) (Ruberte et al., 2004). Patients with proliferative diabetic retinopathy have increased vitreous levels of IGF1 (Boulton et al., 1997; Grant et al., 1986; Meyer‐Schwickerath et al., 1993). Thus, although IGF1 plays a key role in pathological NV in retina, the origin of IGF1 and interaction between endothelial cell and microglia in the retinal microenvironment has not been well defined. Our results support the idea that IGF1 in diabetic retinopathy or retinopathy of prematurity may be derived from retinal microglia. We further suggest that abundant IGF1 produced by activated microglia stabilizes VEGF‐driven retinal neovessels resulting in the exacerbation and prolonged pathological NV in Tgfβr2 KO (ΔMG) OIR.

We have also shown that Tgfβ1‐regulated Igf1 expression using hiPS derived microglia cells and Tgfβ1 rescued Igf1 upregulation under chemically induced hypoxia. We surmised Igf1 upregulation in hypoxic microglia induces retinal NVs synergistically with hypoxia induced Vegfa upregulation. Igf1 upregulation after hypoxia occurred at alter time than Vegfa upregulation, suggesting that Igf1 released by hypoxic microglia plays an important role in stabilizing and prolonging pathological NV induced by Vegfa under hypoxia.

Collectively, these results demonstrate that Tgfβ signaling in retinal microglia is critical for maintaining their homeostatic function and regulation of their hypoxic response in ischemic retinopathy. Targeting Tgfβ signaling in microglia may be a potential therapeutic target to treat pathological NVs in ischemic retinopathy.

## CONFLICT OF INTEREST

The authors declare no conflict of interest.

## Supporting information


**FIGURE S1**: The identification of hiPSCs derived microglia cells (pMG). (a) The expression levels of Cd11b, P2ry12, Tmem119, and Vegfr2 were evaluated in pMG, peripheral blood mononuclear cell (PBMC), and human umbilical vein endothelial cell (HUVEC) (*n* = 4). Data are mean ± SEM. *p* Values were calculated using Ordinary one‐way ANOVA and Tukey's multiple comparison test. *p < .05, ***p < .001, ****p < .0001. (b) Flow cytometry of pMG. CD45, CD14, CX3CR1, and CD11b were expressed in pMG. (c) Immunocytochemistry of pMG. The signals of Cd45, Cd14, IBA1, CCL2, P2RY12, and PU.1 were detected. Blue: DAPI.

**TABLE S1**: Primer sequences for PCR.
**TABLE S2**: Taqman assays for PCR.

## Data Availability

The data that support the findings of this study are available from the corresponding author upon reasonable request.
