# Supplementary figures and images for: Deletion of Tgfβ signal in activated microglia prolongs hypoxia‐induced retinal neovascularization enhancing Igf1 expression and retinal leukostasis
Source: Glia. 2022 May 25;70(9):1762–76. doi: 10.1002/glia.24218 (PMC9540888; doi:10.1002/glia.24218)

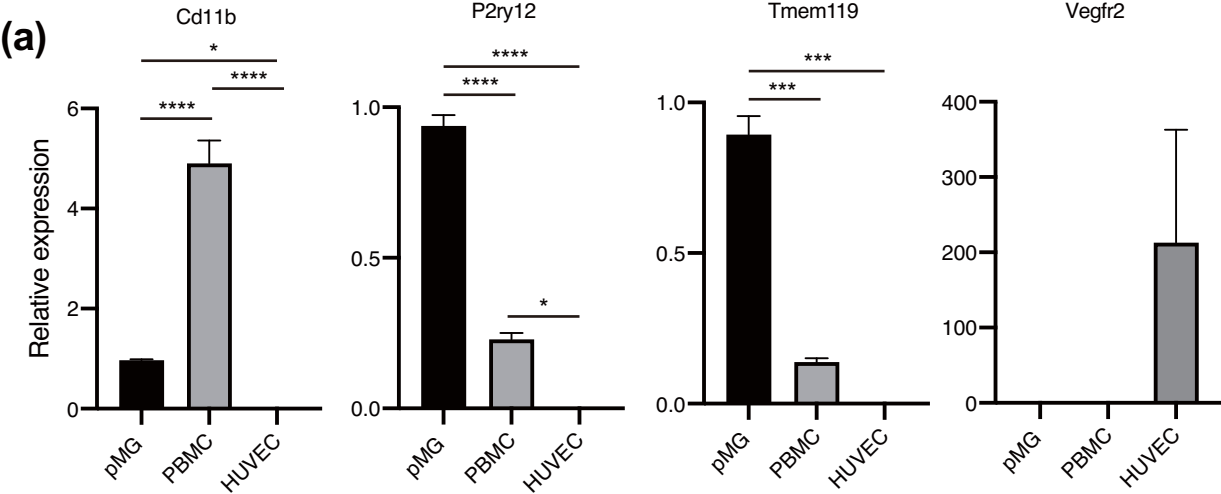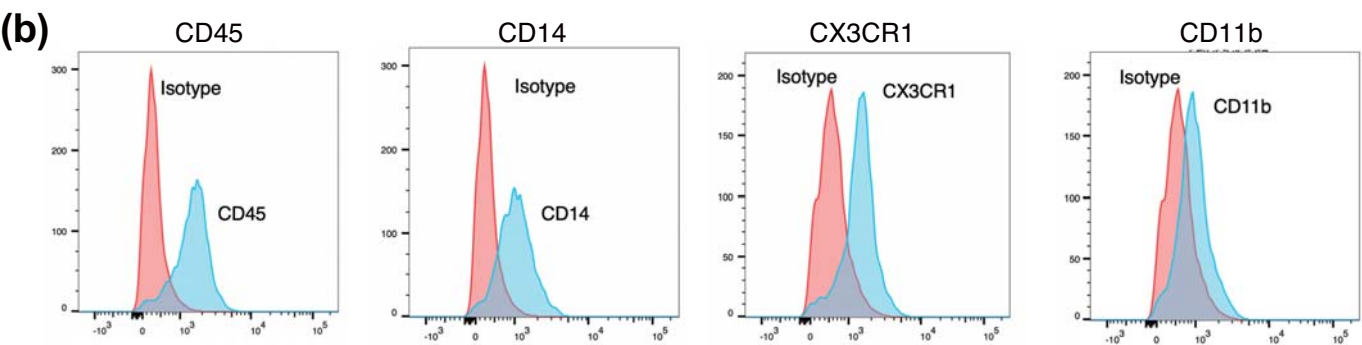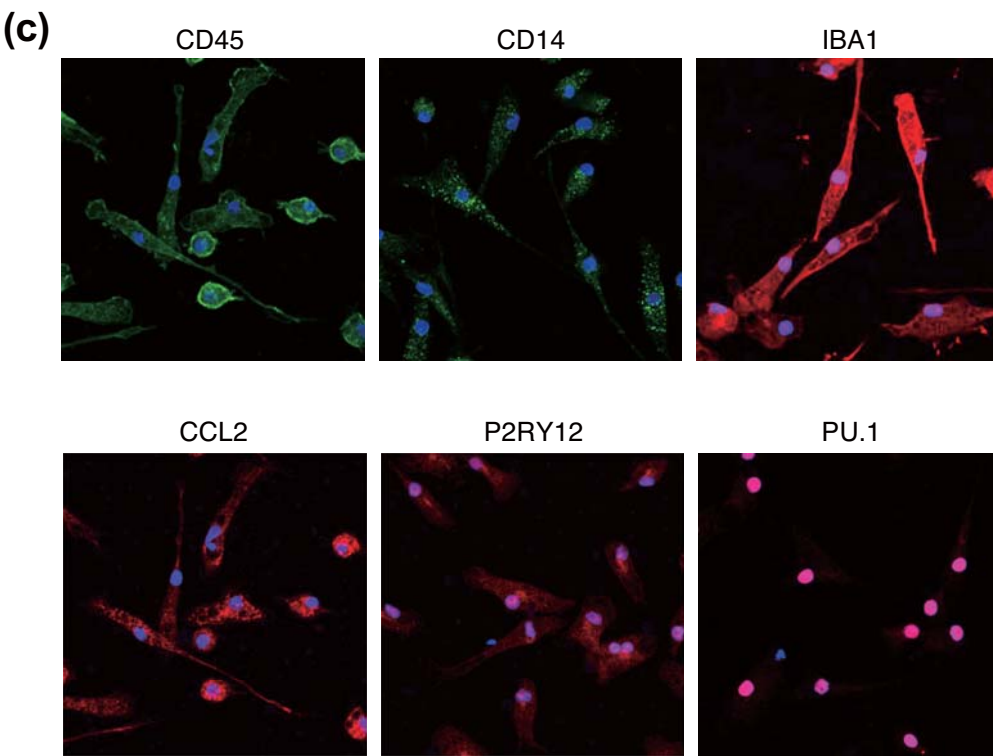

Supplement: Supplementary file 1 — FIGURE S1: The identification of hiPSCs derived microglia cells (pMG). (a) The expression levels of Cd11b, P2ry12, Tmem119, and Vegfr2 were evaluated in pMG, peripheral blood mononuclear cell (PBMC), and human umbilical vein endothelial cell (HUVEC) (n = 4). Data are mean ± SEM. p Values were calculated using Ordinary one‐way ANOVA and Tukey's multiple comparison test. *p < .05, ***p < .001, ****p < .0001. (b) Flow cytometry of pMG. CD45, CD14, CX3CR1, and CD11b were expressed in pMG. (c) Immunocytochemistry of pMG. The signals of Cd45, Cd14, IBA1, CCL2, P2RY12, and PU.1 were detected. Blue: DAPI. [file GLIA-70-1762-s002.pdf]
