# Supplementary material for: Deletion of Tgfβ signal in activated microglia prolongs hypoxia‐induced retinal neovascularization enhancing Igf1 expression and retinal leukostasis
Source: Glia. 2022 May 25;70(9):1762–76. doi: 10.1002/glia.24218 (PMC9540888; doi:10.1002/glia.24218)
Supplement: Supplementary file 2 — TABLE S1: Primer sequences for PCR. TABLE S2: Taqman assays for PCR. [file GLIA-70-1762-s001.docx]

Supplementary Table 1. Primer sequences for PCR.

| Mouse *ActB* forward | AGATCTGGCACCACACCTTC |
| --- | --- |
| Mouse *ActB* reverse | GGGGTGTTGAAGGTCTCAAA |
| Mouse *Ccl2* forward | GCTACAAGAGGATCACCAGCAG |
| Mouse *Ccl2* reverse | GTCTGGACCCATTCCTTCTTGG |
| Mouse *Ccl8* forward | GGGTGCTGAAAAGCTACGAGAG |
| Mouse *Ccl8* reverse | GGATCTCCATGTACTCACTGACC |
| Mouse *Icam1* forward | AAACCAGACCCTGGAACTGCAC |
| Mouse *Icam1* reverse | GCCTGGCATTTCAGAGTCTGCT |
| Mouse *Vcam1* forward | GCTATGAGGATGGAAGACTCTGG |
| Mouse *Vcam1* reverse | ACTTGTGCAGCCACCTGAGATC |
| Mouse *Il6* forward | TACCACTTCACAAGTCGGAGGC |
| Mouse *Il6* reverse | CTGCAAGTGCATCATCGTTGTTC |
| Mouse *Il1b* forward | TGGACCTTCCAGGATGAGGACA |
| Mouse *Il1b* reverse | GTTCATCTCGGAGCCTGTAGTG |
| Mouse *Tnfa* forward | GGTGCCTATGTCTCAGCCTCTT |
| Mouse *Tnfa* reverse | GCCATAGAACTGATGAGAGGGAG |
| Mouse *Igf1* forward | GTGGATGCTCTTCAGTTCGTGTG |
| Mouse *Igf1* reverse | TCCAGTCTCCTCAGATCACAGC |
| Mouse *Vegfa* forward | AACGATGAAGCCCTGGAGT |
| Mouse *Vegfa* reverse | AGGTTTGATCCGCATGATCT |
| Mouse *Fgf2* forward | AAGCGGCTCTACTGCAAGAACG |
| Mouse *Fgf2* reverse | CCTTGATAGACACAACTCCTCTC |
| Mouse *Tgfb1* forward | TGATACGCCTGAGTGGCTGTCT |
| Mouse *Tgfb1* reverse | CACAAGAGCAGTGAGCGCTGAA |
| Mouse *Tgfbr1* forward | TGCTCCAAACCACAGAGTAGGC |
| Mouse *Tgfbr1* reverse | CCCAGAACACTAAGCCCATTGC |
| Mouse *Tgfbr2* forward | CCTACTCTGTCTGTGGATGACC |
| Mouse *Tgfbr2* reverse | GACATCCGTCTGCTTGAACGAC |
| Mouse *Tgfbr2-E3* forward | CTCTGGAGACGGTTTGCCAC |
| Mouse *Tgfbr2-E3* reverse | AGATGATGTAATCGTTGCACTC |
| Mouse *Tgfbr2-E4* forward | GACCTGTTGTTGGTCATTATCC |
| Mouse *Tgfbr2-E4* reverse | CGTCCTCCAGGATGATGGC |
| Human *ActB* forward | CACCAACTGGGACGACAT |
| Human *ActB* reverse | ACAGCCTGGATAGCAACG |
| Human *Igf1* forward | CTCTTCAGTTCGTGTGTGGAGAC |
| Human *Igf1* reverse | CAGCCTCCTTAGATCACAGCTC |
| Human *Cd11b* forward | GGAACGCCATTGTCTGCTTTCG |
| Human *Cd11b* reverse | ATGCTGAGGTCATCCTGGCAGA |
| Human *P2ry12* forward | TGCCAAACTGGGAACAGGACCA |
| Human *P2ry12* reverse | TGGTGGTCTTCTGGTAGCGATC |
| Human *Tmem119* forward | GGATAGTGGACTTCTTCCGCCA |
| Human *Tmem119* reverse | GGAAGGACGATGGGTAATAGGC |
| Human *Vegfr2* forward | GGAACCTCACTATCCGCAGAGT |
| Human *Vegfr2* reverse | CCAAGTTCGTCTTTTCCTGGGC |

Supplementary Table 2. Taqman assays for PCR.

| *ActB* | Mm02619580_g1 |
| --- | --- |
| *Vegfa* | Mm00437306_m1 |
| *Epo* | Mm01202755_m1 |
| *Tnfa* | Mm00443258_m1 |
| *Ccl2* | Mm00441242_m1 |
| *Ccl3* | Mm00441259_g1 |
| *Il1b* | Mm00434228_m1 |
| *Cited2* | Mm01188099_g1 |
